# Discoidin domain receptor 1 promotes Th17 cell migration by activating the RhoA/ROCK/MAPK/ERK signaling pathway

**DOI:** 10.18632/oncotarget.10455

**Published:** 2016-07-06

**Authors:** Mohammed-Amine El Azreq, Maleck Kadiri, Marc Boisvert, Nathalie Pagé, Philippe A. Tessier, Fawzi Aoudjit

**Affiliations:** ^1^ Axe de Recherche sur les Maladies Infectieuses et Immunitaires, Centre de Recherche du Centre Hospitalier Universitaire de Québec, Québec, QC, Canada; ^2^ Département de Microbiologie-Immunologie, Faculté de Médecine, Université Laval, Québec, QC, Canada

**Keywords:** Th17 cells, migration, DDR1, 3D collagen, RhoA/ROCK, Immunology and Microbiology Section, Immune response, Immunity

## Abstract

Effector T cell migration through the tissue extracellular matrix (ECM) is an important step of the adaptive immune response and in the development of inflammatory diseases. However, the mechanisms involved in this process are still poorly understood. In this study, we addressed the role of a collagen receptor, the discoidin domain receptor 1 (DDR1), in the migration of Th17 cells. We showed that the vast majority of human Th17 cells express DDR1 and that silencing DDR1 or using the blocking recombinant receptor DDR1:Fc significantly reduced their motility and invasion in three-dimensional (3D) collagen. DDR1 promoted Th17 migration by activating RhoA/ROCK and MAPK/ERK signaling pathways. Interestingly, the RhoA/ROCK signaling module was required for MAPK/ERK activation. Finally, we showed that DDR1 is important for the recruitment of Th17 cells into the mouse dorsal air pouch containing the chemoattractant CCL20. Collectively, our results indicate that DDR1, *via* the activation of RhoA/ROCK/MAPK/ERK signaling axis, is a key pathway of effector T cell migration through collagen of perivascular tissues. As such, DDR1 can contribute to the development of Th17-dependent inflammatory diseases.

## INTRODUCTION

T cell migration through ECM in peripheral tissues is critical for the adaptive immune response and in the development of inflammation. To reach their target sites and exert their functions, activated/effector T cells leave the lymph nodes, enter the circulation, then the perivascular tissues through a process known as trans-endothelial extravasation [1, 2]. There, effector T cells also migrate through the ECM of interstitial tissues in which collagen type I (collagen) is the most abundant matrix [3, 4].

The use of 3D collagen models such as collagen gels, which are more relevant physiologically than the 2D models, revealed that cells could use different modes of migration. Cells such as fibroblasts and cancer cells use the mesenchymal-type mode of migration, which is dependent on strong adhesive forces mediated by integrins, stress fiber formation and ECM remodeling by matrix metalloproteinases (MMPs) [5, 6]. However, activated T cells, dendritic cells and monocytes migrate in 3D collagen and in interstitial tissues using the amoeboid movement, relying on contact guidance and squeezing through existing gaps, independently from integrins and MMPs [7-10]. These studies suggest that additional receptors are likely to stimulate the amoeboid movement of leukocytes in 3D collagen.

Discoidin domain receptors (DDR1 and DDR2) are non-integrin tyrosine kinase transmembrane receptors that bind different types of collagen [11, 12]. DDR1 is expressed in many epithelial and carcinoma cells and has been associated with cancer cell invasion in 3D collagen mainly through the promotion of MMP production [13]. In this context, it has recently been reported that DDR1 enhances the formation of invadosomes and MMP activity in cancer cells *via* the small GTPase Cdc42 [14]. DDR1 also stimulates the collective migration of cancer cells *via* the Giα13 pathway [15, 16]. In addition to epithelial and carcinoma cells, short-term activated human T cells also express DDR1 [17-19] and the blocking recombinant receptor DDR1:Fc reduces their migration across collagen gel-coated transwells [18]. Moreover, DDR1 overexpression enhances THP-1 monocytic cell line migration in 3D collagen [19]. Despite these findings, the extent to which DDR1 promotes migration of amoeboid cells such as effector T cells in 3D collagen is still poorly understood.

Th17 are a subpopulation of T helper cells that are specialized in the production of IL-17. They play important roles in anti-microbial immunity [20], autoimmune diseases [21-23], and have been implicated in tumor growth and anti-cancer immunity [24]. Therefore, it is critical to understand how Th17 cells migrate through the tissue ECM.

In this study, we show that DDR1 is expressed in human Th17 cells and that it is involved in their migration in 3D collagen by activating the small GTPase RhoA and its effector Rho-associated kinase (ROCK) and the MAPK/ERK pathways. Blocking Th17 interactions with collagen using DDR1:Fc reduced the recruitment of Th17 cells into the mouse air pouch containing the chemoattractant CCL20. Together, these results indicate that DDR1 is a critical mediator of Th17 migration through collagen of perivascular tissues.

## RESULTS

### Human Th17 cells express DDR1

**Figure 1:**
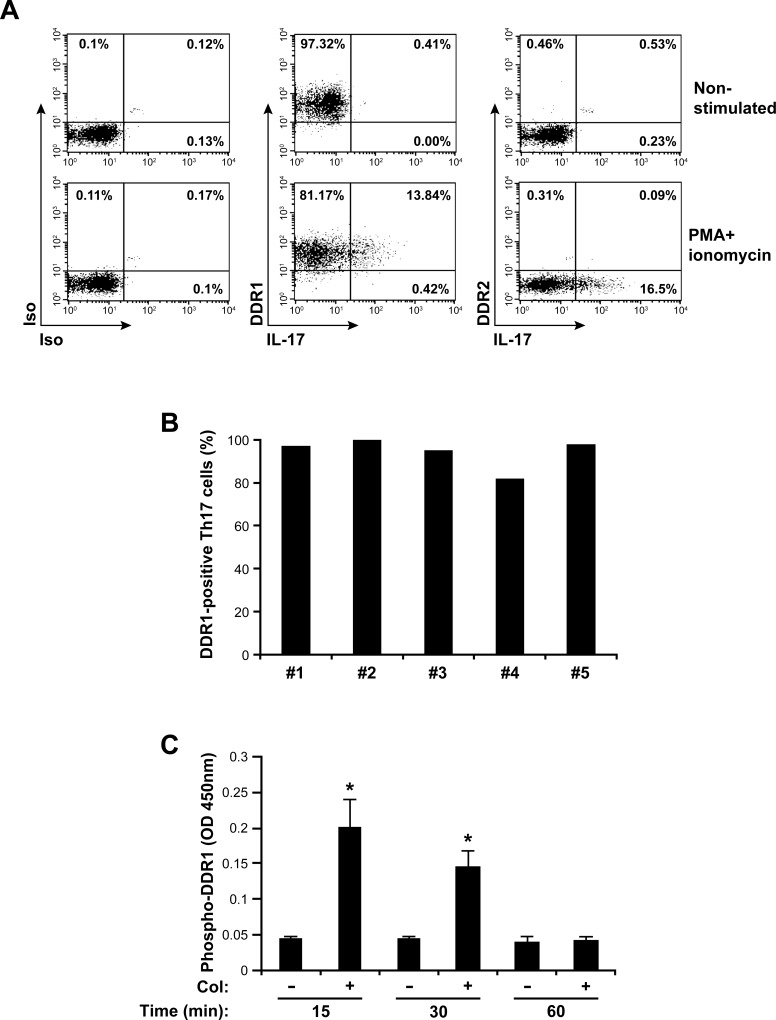
DDR1 is expressed and is functional in human Th17 cells **A.** Polarized human Th17 cells were activated or not with PMA+ionomycin for 6 hours in the presence of brefeldin A. The cells were washed and stained with antibodies against DDR1 and DDR2 and with anti-IL-17 mAb to identify IL-17-producing cells as described under the “Materials and Methods” section. Staining with isotypic antibodies (Iso) were used as controls. The cells were then analyzed by flow cytometry. The FACS plots are representative of five different samples. **B.** The histogram represents percentages of human Th17 cells expressing DDR1 from five different blood donors. **C.** Collagen induces DDR1 tyrosine phosphorylation. The cells were cultured on plastic (−) or in collagen gels (+) for 15, 30 or 60 min. The cells were lysed and DDR1 tyrosine phosphorylation levels were determined by ELISA assay. The results are mean values ± SD of five independent experiments performed with polarized Th17 cells derived from five different blood donors. *p < 0.05.

We have previously shown that DDR1 expression is induced in human CD4^+^ T cells upon their activation through the T cell receptor [18, 25]. Here, we analyzed the expression of DDR1 and DDR2 in human Th17 effector cells. We found that almost all polarized Th17 cells express DDR1 but not DDR2 (Figure 1A). To confirm that IL-17-producing cells (Th17 cells) express DDR1, we activated human polarized Th17 cells with PMA+ionomycin to induce IL-17 production, and we determined the expression of DDRs. Flow cytometry analysis showed that the vast majority of Th17 cells express DDR1 but not DDR2 (Figure 1A). Expression analysis on human Th17 cells polarized from five different blood donors showed that between 80-100% of human Th17 cells express DDR1 (Figure 1B). These results indicate that human Th17 cells preferentially express DDR1. In addition, DDR1 is activated by 3D collagen in human polarized Th17 cells. The results showed that collagen gel induced a rapid tyrosine phosphorylation of DDR1 with a peak at 15 minutes of stimulation, which returns to baseline after 1 h (Figure 1C). This DDR1 tyrosine phosphorylation kinetic is consistent with that observed with cells growing in suspension such as K562 [26] and B cell lymphoma [27]. Thus, DDR1 is expressed and is functional in human Th17 cells.

### DDR1 is involved in Th17 migration in 3D collagen

**Figure 2:**
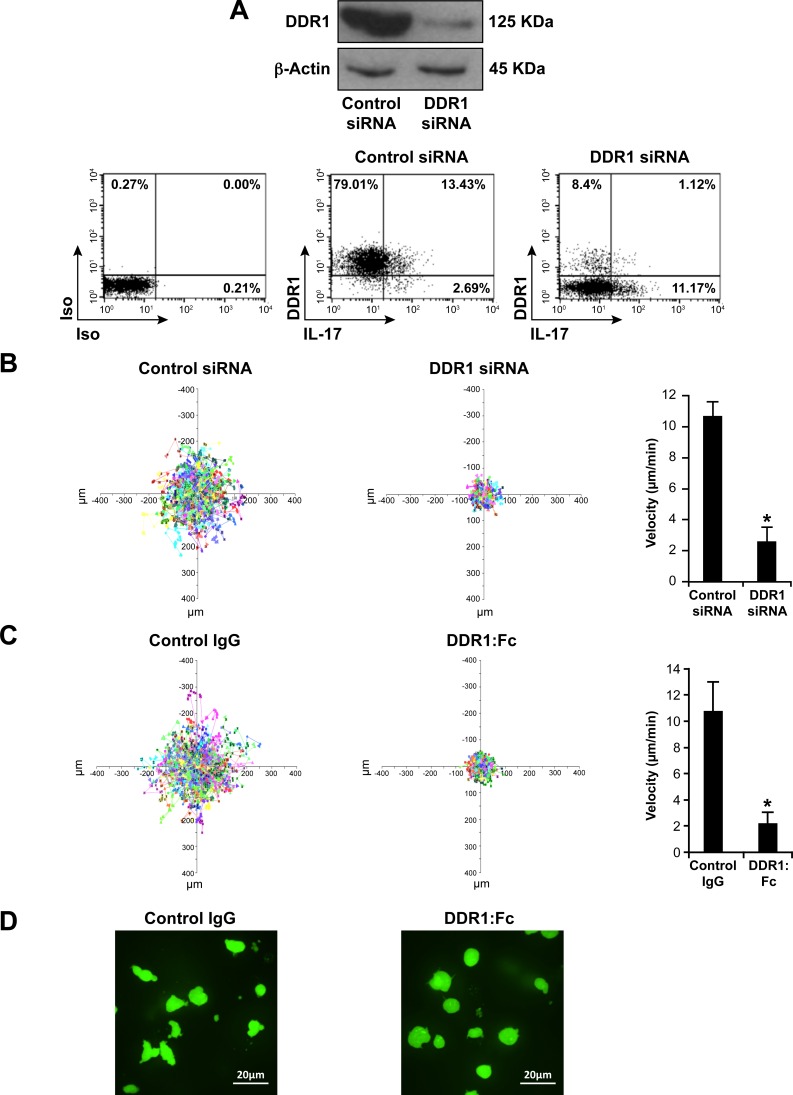
DDR1 promotes human Th17 motility in 3D collagen **A.** DDR1 expression is reduced by specific DDR1 siRNA (HSS1878780). Polarized Th17 cells were transfected with control or with DDR1 siRNAs and DDR1 expression was assessed by western blot using the anti-DDR1 antibody (C-20). The blot was stripped and reprobed with anti-β-actin antibody to ensure equal loading (top panel). After transfection, the cells were activated with PMA+ionomycin in the presence of brefeldin A to identify IL-17-producing cells, stained with anti-DDR1 and anti-IL-17 antibodies and analyzed by flow cytometry (lower panel). Immunoblot and FACS plots are representative of five independent experiments performed with polarized Th17 cells derived from five different blood donors. **B.** DDR1 siRNA inhibits Th17 motility in 3D collagen. After transfection, the cells were labelled with calcein AM and embedded in collagen gels. Cell migration was evaluated by live cell confocal microscopy and quantified by computer-assisted cell tracking as described in the “Materials and Methods*”* section. Representative cell migration tracks over 30 min are presented as x-y projections (distance, in μm) (left panel). The histogram (right panel) represents the mean velocity of 100 cells presented as μm/min. **C.** DDR1:Fc inhibits Th17 motility in 3D collagen. The cells were embedded in collagen gels containing control human IgG (Fc fragment) or human DDR1:Fc recombinant proteins and cell motility was determined as above. Results (B and C right panels) are mean values ± SD of five independent experiments performed with polarized Th17 cells derived from five different blood donors. *p < 0.05. **D.** DDR1:Fc inhibits the migratory shape of polarized Th17 cells. Representative photography images from five different experiments of polarized Th17 cells migrating in collagen gels containing either control IgG or DDR1:Fc (400X magnification).

To determine the role of DDR1 in Th17 migration, we first used an RNAi approach and studied Th17 cell motility in 3D collagen by live cell confocal microscopy. DDR1 specific siRNA (HSS1878780) but not control siRNA reduced total DDR1 protein levels by 80% (western blot) and DDR1 surface expression by 90% (FACS) (Figure 2A). DDR1 specific siRNA had no effect on IL-17 production (Figure 2A, lower panel) and on the levels of the CD3 receptor complex (Figure S1), thus supporting the specificity of DDR1 silencing. Polarized Th17 cells that have been transfected with DDR1 siRNA exhibited a dramatic decrease in motility when compared to cells transfected with control siRNA (Figure 2B) and (Video S1). Quantification analysis shows a 75% reduction in the velocity of DDR1 siRNA-transfected cells (Figure 2B, right panel). Similar results were obtained with an additional DDR1 siRNA sequence (HSS187879) (Figure S2).

As a complementary approach to siRNA, we used the blocking recombinant human receptor DDR1:Fc, which has been shown to block DDR1-mediated cell interactions with collagen [18, 28, 29]. We found that inclusion of recombinant human DDR1:Fc in the collagen gel reduced the motility of polarized human Th17 cells by 80% when compared to cells migrating in collagen gels containing control recombinant human IgG (Fc fragment) (Figure 2C). However, DDR1:Fc did not affect neutrophil migration in 3D collagen (Figure S3), which has been shown to be dependent on DDR2 [30], indicating the specificity of DDR1:Fc in inhibiting DDR1. The DDR1:Fc also inhibited the roundish/ellipsoid migratory shape of Th17 cells (Figure 2D), which characterizes the amoeboid movement of activated T cells in 3D collagen [31]. The pro-migratory role of DDR1 in Th17 cells is further supported by the fact that Th17 cells released from collagen gels containing DDR1:Fc adhered more to collagen than those released from control collagen gels (Figure S4). This suggests that DDR1 promotes Th17 migration by reducing firm adhesion to collagen, which is consistent with the amoeboid movement of activated T cells, which occurs independently from strong adhesive forces.

### DDR1 promotes Th17 invasion and chemotaxis in 3D collagen

**Figure 3:**
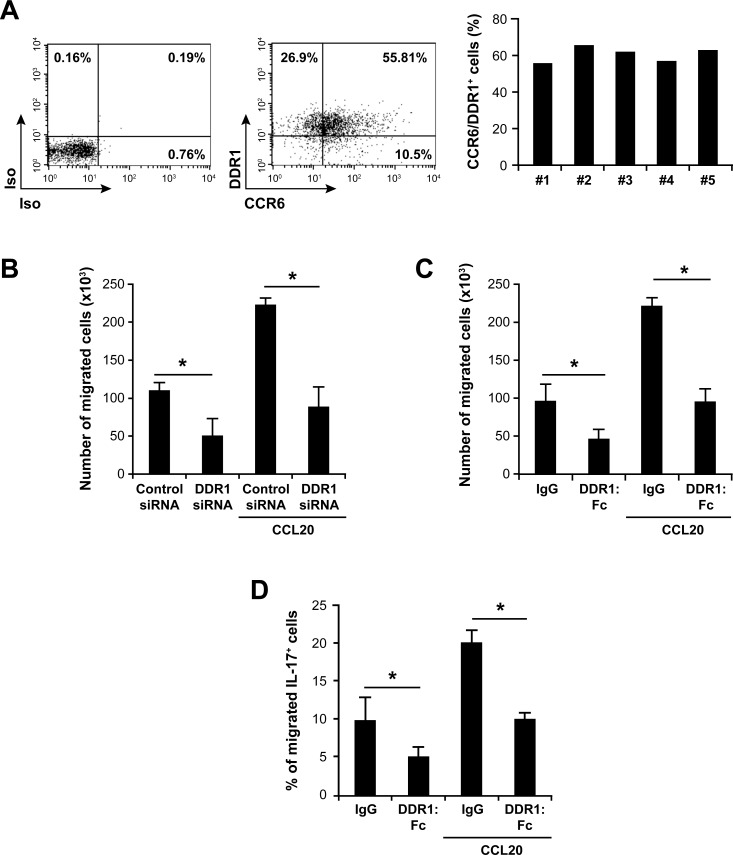
DDR1 promotes invasion and chemotaxis of human Th17 cells in 3D collagen **A.** Polarized human Th17 cells co-express CCR6 and DDR1. The cells were stained with anti CCR6 and anti-DDR1 or with control isotypic (Iso) antibodies and analyzed by flow cytometry. The FACS plots are representative of five different samples (left panel). The histogram represents percentages of polarized Th17 cells co-expressing CCR6 and DDR1 derived from five different blood donors. **B.** DDR1 silencing reduces invasion and CCL20-directed migration of polarized Th17 cells in 3D collagen. The cells transfected with DDR1 or control siRNAs were added on top of collagen gel-coated transwells. After 24 h, cells that had passed to the other side of the transwells and in the outer wells, which contained X-vivo medium with or without 1 μg/ml of CCL20 were recovered and counted microscopically. **C.** Effect of DDR1:Fc on invasion and on CCL20-directed migration of Th17 cells through 3D collagen. Cells were added on the top of collagen gels containing recombinant human DDR1:Fc or control IgG (Fc fragment) proteins. After 24 h, cells that had passed to the outer wells were counted. **D.** Effect of DDR1:Fc on invasion and CCL20-directed migration of IL-17-producing cells. Cells that have migrated through collagen gel-coated transwells were recovered from the lower chambers and activated with PMA+ionomycin in the presence of brefeldin A to identify IL-17-producing cells. The cells were fixed/permeabilized, stained with anti-IL-17-Alexa-647 mAb and analyzed by flow cytometry. Results (panels B, C and D) are mean values ± SD of five independent experiments performed with Th17 cells derived from five different blood donors. *p < 0.05.

Then, we examined the role of DDR1 in invasion and chemotaxis of Th17 cells using collagen gel-coated transwells. Th17 cells express the CCR6 receptor [32] and respond to the chemokine CCL20 [32-36]. We found that DDR1 and CCR6 are co-expressed on 55%-65% of the polarized Th17 cells (Figure 3A). These cells migrated through collagen gel-coated transwells and the presence of CCL20 caused a 2.5 fold increase of their migration (Figure 3B and 3C). Transfection of DDR1 siRNA (Figure 3B) or inclusion of DDR1:Fc in the collagen gels (Figure 3C) reduced Th17 invasion by 50-60% both in the absence and the presence of CCL20. Unlike neutrophil chemotaxis in 3D collagen [30], the pan-MMP inhibitor GM6001 had no effect on Th17 migration and chemotaxis (Figure S5), suggesting that MMPs are not required.

To confirm that migration of IL-17-producing cells in 3D collagen is affected upon DDR1 inhibition, we determined the number of IL-17-producing cells that migrated through collagen gel-coated transwells. To this end, the recovered cells from the outer wells were activated with PMA+ionomycin and analyzed for their production of IL-17 by flow cytometry. The data indicate that the presence of DDR1:Fc reduced the number of Th17 cells migrating randomly and in response to CCL20 (Figure 3D). Although CCR6 is expressed on 55-65% of the polarized Th17 cells, only 20% of IL-17-postive cells migrated towards CCL20 (maximum observed). This is in line with the fact that not all CCR6-positive cells produce IL-17 [37]. Taken together, these results demonstrate an important role for DDR1 in human Th17 cell migration in 3D collagen.

### DDR1-induced Th17 migration involves the RhoA/ROCK pathway

**Figure 4:**
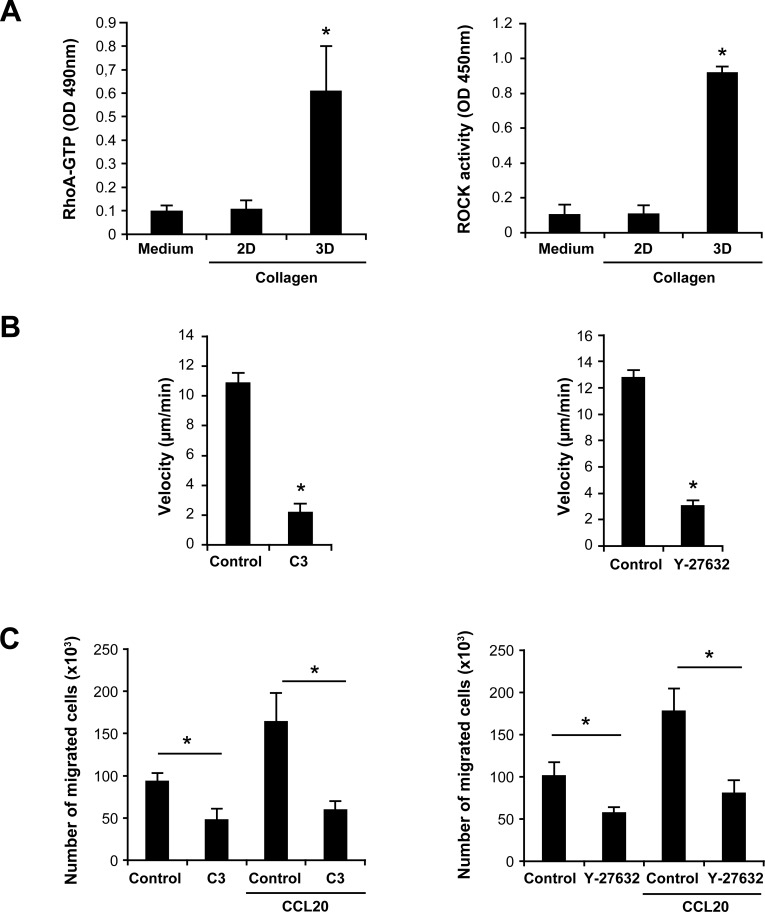
Th17 migration in 3D collagen involves the RhoA/ROCK signaling pathway **A.** 3D collagen increases RhoA and ROCK activation. Th17 cells were cultured on plastic (medium), on monomeric immobilized collagen (2D) or in collagen gels (3D) for 1 h. The cells were released from collagen gels after a collagenase treatment and equal cell numbers were lysed in RIPA buffer. GTP-bound RhoA levels and ROCK activity were measured using specific RhoA G-LISA and ROCK ELISA, respectively. **B.** RhoA/ROCK inhibitors reduce Th17 cell migration. Th17 cells were incubated or not for 1 h with diluent (control) or with 1 μg/ml of the RhoA inhibitor (C3 toxin) or with 30 μM of the ROCK inhibitor (Y-27632) and embedded in collagen gels. Cell migration was evaluated by live cell confocal microscopy and quantified by computer-assisted cell tracking as described in the “Materials and Methods” section. **C.** RhoA/ROCK inhibitors inhibit CCL20-directed migration of Th17 cells in 3D collagen. The cells were incubated as above with the RhoA/ROCK inhibitors and tested for their invasion capacity of collagen gel-coated transwells. Cells that had passed to the outer wells containing medium alone or with CCL20 were then counted microscopically. Results (panels A, B and C) are mean values ± SD of three independent experiments performed with Th17 cells derived from three different blood donors. *p < 0.05.

**Figure 5:**
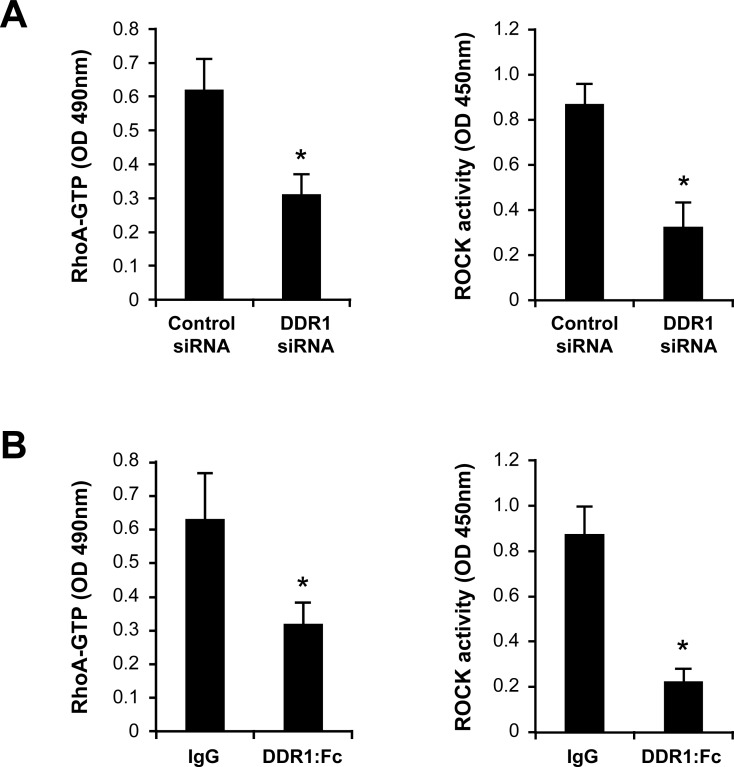
Inhibition of DDR1 decreases RhoA/ROCK activity in Th17 cells cultured in 3D collagen **A.** Effect of DDR1 siRNA on collagen gel-induced RhoA/ROCK activation. Cells were transfected with control or DDR1 siRNAs and embedded in collagen gels for 1 h. The cells were released from collagen, lysed and GTP-bound RhoA and ROCK activity were determined. **B.** Effect of DDR1:Fc on RhoA/ROCK activity. The cells were embedded in collagen gels containing control recombinant human IgG (Fc fragment) or DDR1:Fc proteins and after 1 h, RhoA and ROCK activities were determined. Results are mean values ± SD of three independent experiments performed with Th17 cells derived from three different blood donors. *p < 0.05.

We then investigated the mechanisms by which DDR1 promotes Th17 migration in 3D collagen. The Rho family of small GTPases are important signaling molecules in cell migration but their role in T cell movement in 3D collagen has not been previously addressed. Thus, we examined the implication of RhoA and of its effector, the kinase ROCK. We found that only collagen gel (3D) but not coated monomeric collagen (2D) activated RhoA and ROCK (Figure 4A). The use of collagenase to release the cells from collagen had no effect on RhoA or ROCK activity (data not shown). To determine the implication of RhoA and ROCK in Th17 migration, we used specific inhibitors, namely the C3 toxin (RhoA inhibitor) and Y27632 (ROCK inhibitor). Both inhibitors strongly decreased Th17 motility (Figure 4B) as well as Th17 invasion and chemotaxis in collagen gel-coated transwells (Figure 4C). Although CCL20 caused a two-fold increase of RhoA activation, it had no additional or inhibitory effect in cells stimulated with collagen gel (data not shown). We then asked whether DDR1 inhibition would impact RhoA/ROCK activation. We found that DDR1 siRNA and DDR1:Fc decreased RhoA activation by 50% and that of ROCK by 65-75% in Th17 cells cultured in collagen gels (Figure 5A and 5B).

Since the small GTPase Rac1 can inhibit the amoeboid movement [38, 39], we verified its implication in Th17 migration. We found that collagen gel reduces Rac1 activity and blocking DDR1 restored its activity (Figure S6). In addition, the Rac1 inhibitor enhanced Th17 migration. Taken together, these data indicate that increased activation of RhoA/ROCK and decreased Rac1 activity, through DDR1, are important for Th17 migration in 3D collagen.

### DDR1-induced Th17 migration involves the MAPK/ERK pathway

3D collagen has been associated with the activation of MAPK/ERK, which is an important pathway in cell migration. We therefore examined its implication in human Th17 migration.

**Figure 6:**
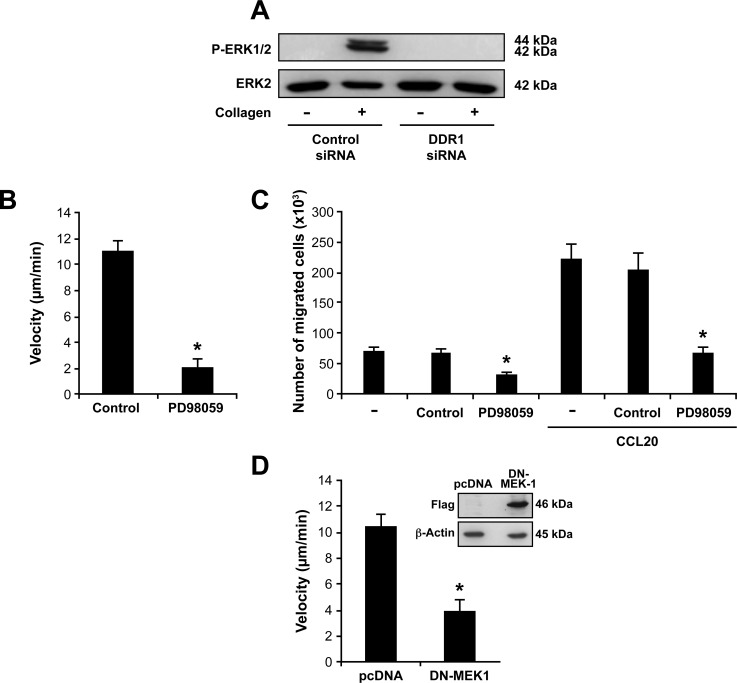
DDR1 promotes Th17 migration by activating the MAPK/ERK pathway **A.** DDR1 silencing inhibits collagen gel-induced ERK1/2 phosphorylation. The cells were transfected with DDR1 or control siRNAs, embedded in collagen gels for 1 h and then ERK1/2 phosphorylation was determined by western blot. The blot was stripped and reprobed with anti-ERK2 to ensure equal loading. **B.**-**C.** The MAPK/ERK inhibitor reduced Th17 cell migration in 3D collagen. The cells were cultured alone (−) or with the MAPK/ERK inhibitor PD98059 (20 μM) or diluent (control) for 1 h and then tested for their capacity to migrate. Cell migration in 3D collagen was evaluated by live cell confocal microscopy **B.** and by invasion of collagen gel-coated transwells **C.**. **D.** Dominant-negative form of MEK-1 (DN-MEK-1) reduces Th17 cell migration in collagen. The cells were transfected with control pcDNA and DN-MEK-1 plasmids and their migration was determined by confocal microscopy. The western blot shows the expression of DN-MEK-1 (flag-tagged) in control and in DN-MEK-1-transfected cells. Results (panels B, C and D) are mean values ± SD of three independent experiments performed with Th17 cells derived from three different blood donors. *p < 0.05.

After 1 h of stimulation, collagen gel increased ERK phosphorylation and this effect was mediated *via* DDR1 since it was abolished in DDR1 siRNA-transfected cells (Figure 6A). Similar results were obtained when the cells were cultured in collagen gels for 2 or 3 h (Data not shown). In addition, the MAPK/ERK inhibitor PD98059 strongly reduced Th17 cell motility (Figure 6B) and invasion (Figure 6C) in collagen gels, and expression of a dominant-negative form of MEK-1 (DN-MEK-1) also reduced Th17 migration (Figure 6D). Together, these data support the implication of the MAPK/ERK pathway in Th17 migration in 3D collagen.

**Figure 7:**
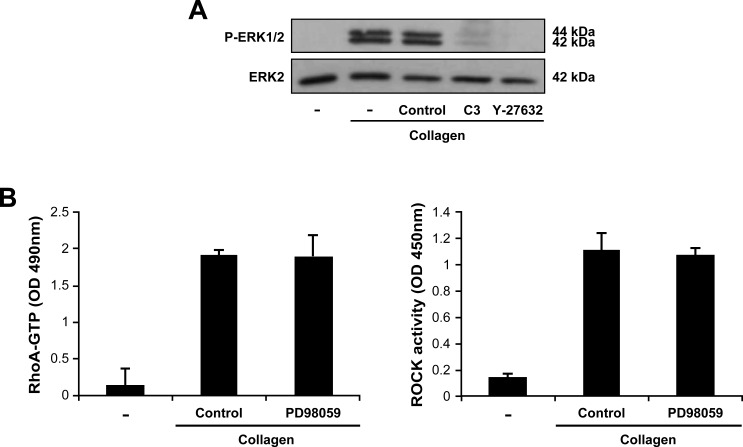
The MAPK/ERK is downstream of RhoA/ROCK in Th17 cells **A.** RhoA and ROCK are necessary for 3D collagen-induced ERK activation. The cells were incubated for 1 h in medium alone (−) or in medium containing C3 toxin or Y-27632 inhibitors. The cells were then embedded in collagen gels for 1 h, after which, ERK1/2 phosphorylation was determined by western blot. The results are representative of three independent experiments. **B.** The MAPK/ERK inhibitor had no effect on 3D collagen-induced RhoA and ROCK activation. The cells were cultured for 1 h in medium alone (−) or in medium containing the MAPK/ERK inhibitor PD98059 (20 μM) or diluent (control) and then embedded in collagen gels. After 1 h, RhoA and ROCK activities were measured by ELISA assays. Results are mean values ± SD of three independent experiments performed with Th17 cells derived from three different blood donors.

We also found that ERK activation was dependent on the RhoA/ROCK pathway since the C3 and Y27632 inhibitors abolished the ability of collagen gel to phosphorylate ERK in Th17 cells (Figure 7A). However, the PD98059 inhibitor had no effect on RhoA/ROCK activation (Figure 7B) indicating that ERK is downstream of RhoA/ROCK. Therefore, DDR1 promotes human Th17 migration in 3D collagen by activating the RhoA/ROCK/MAPK/ERK signaling axis.

### DDR1 promotes Th17 cell migration *in vivo*

**Figure 8:**
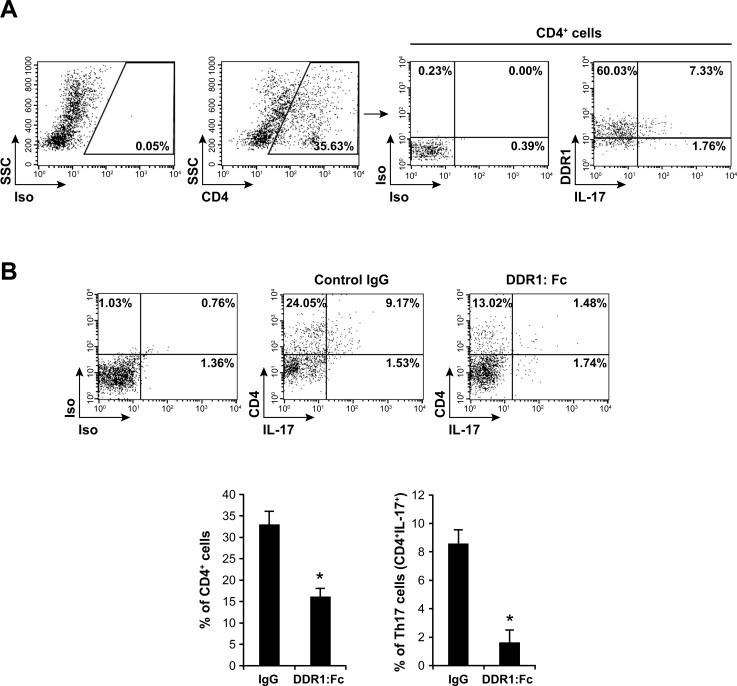
DDR1 promotes Th17 migration *in vivo* **A.** Th17 cells recruited into mouse dorsal air pouch express DDR1. Air pouches were raised as described in the “Materials and Methods” section. The cells were recovered from air pouches 24 h after the injection of CCL20 and were activated with PMA+ionomycin in the presence of brefeldin A to identify IL-17-positive cells. The cells were stained with anti-CD4, then with intracellular anti-DDR1 and anti-IL-17 antibodies and analyzed by flow cytometry. Staining with isotypic antibodies (Iso) were used as controls. DDR1/IL-17-positive cells in the right panel are gated on the CD4 positive population. The FACS plots are representative of ten mice (*n* = 10). **B.** DDR1:Fc inhibits Th17 recruitment into the air pouch. Mouse recombinant DDR1:Fc or control IgG (Fc fragment) proteins were injected into the air pouches at the same time as CCL20. After 24 h, the cells were recovered, activated with PMA+ionomycin in the presence of brefeldin A, stained with anti-CD4 and anti-IL-17 antibodies and analyzed by flow cytometry. The FACS plots are representative of ten mice. The histogram represents mean values ± SD (*n* = 10) of CD4^+^ and CD4^+^/IL-17^+^ (Th17) cells recovered from control IgG- or DDR1:Fc-treated air pouches. *p < 0.05.

To determine the role of DDR1 *in vivo*, we used the mouse dorsal s.c air pouch model of leukocyte migration. In this model, Th17 cells can be recruited into the air pouch in response to the chemokine CCL20 [36]. We found that IL-17^+^ cells represents 9% of the total CD4^+^ T cell population recruited into the air pouch and that DDR1 is expressed in 80% of the recruited Th17 cells (Figure 8A). We then tested whether inhibiting DDR1 activity might influence Th17 recruitment. To this end, we injected the air pouches with blocking recombinant mouse DDR1:Fc receptor or with control recombinant mouse IgG (Fc fragment only), and the cells that had migrated in response to CCL20 after 24 h were analyzed by flow cytometry. We found that DDR1:Fc inhibited the migration of CD4^+^ T cells by almost 60% and that of Th17 cells (IL-17-producing CD4^+^ cells) by almost 85% (Figure 8B). These results demonstrate that DDR1 is involved in Th17 migration in perivascular tissue.

## DISCUSSION

In this study, we report that the collagen receptor DDR1 plays a key role in Th17 cell migration. We showed that DDR1 promotes the motility and invasion of Th17 cells in 3D collagen and in perivascular tissues *in vivo*. In addition, DDR1 enhances Th17 migration by activating the RhoA/ROCK/MAPK/ERK signaling pathway.

DDR1 and DDR2 are widely expressed receptors and are considered to be collagen sensors. They play important roles in cell proliferation, adhesion and migration [11, 12]. Our results showed that human Th17 cells preferentially express DDR1 and inhibition of its activity (siRNA and DDR1:Fc) strongly reduced human Th17 motility, invasion and chemotaxis in 3D collagen. This is associated with the loss of the amoeboid morphology of the migrating cells and with their increased adhesion to collagen suggesting that DDR1 promotes Th17 migration by reducing firm adhesion to collagen. Along these lines, although DDR1 enhances migration of various cancer cells by increasing adhesion to collagen [40-42], it does not always promote cell adhesion. DDR1 binding to collagen inhibits MDCK epithelial cell adhesion to collagen [43] and spreading of NIH-3T3 and MDA-MB-231 cells [44], while promoting their migration. Moreover, and in contrast to DDR2, DDR1 is unable to consistently mediate firm adhesion to collagen [45]. Thus, in cells that migrate using the amoeboid movement such as effector T cells, which rely on low adhesive forces, DDR1 binding to collagen might lessen strong attachment; thereby allowing cell movement. The mechanisms by which DDR1 could reduce firm adhesion are unclear but, as seen in MDCK cells [43], it can inhibit integrin pathways, which mediate strong adhesion to collagen.

Activation of the small GTPase RhoA and its effector ROCK have been involved with the amoeboid movement [46-48]. Herein, we found that 3D collagen increases RhoA and ROCK activation in a DDR1-dependent manner, and inhibition studies indicated that the RhoA/ROCK pathway is critical for Th17 migration in 3D collagen. Concomitantly, 3D collagen reduced Rac1 activity, also in a DDR1-dependent manner, and inhibiting Rac1 activity enhanced Th17 migration in 3D collagen. This is consistent with previous findings indicating that Rac1 promotes cell elongation and mesenchymal-type of migration, and inhibits the amoeboid movement [38, 39]. Thus, Th17 migration in 3D collagen is associated, through DDR1, with increased RhoA/ROCK and decreased Rac1 activity.

In addition to Rho GTPases, we showed that DDR1-induced Th17 migration is dependent on MAPK/ERK activity, which is an important pathway in cell migration. We found that 3D collagen increases ERK phosphorylation in Th17 cells *via* DDR1 and that RhoA and ROCK lie upstream of MAPK/ERK activation. In contrast to our findings, ERK has recently been shown to phosphorylate and activate RhoA [49]. In addition, Annexin-A1 increases breast cancer invasion by activating RhoA in an ERK-dependent fashion. However, in this model, RhoA inhibition also reduced ERK phosphorylation [50] suggesting that the two pathways can influence each other. Based on our data, we propose that DDR1 promotes the amoeboid movement of human Th17 cells in 3D collagen by activating the RhoA/ROCK/MAPK/ERK signaling axis. It is not clear whether RhoA/ROCK stimulates Th17 migration independently from ERK. However, this is plausible since both ERK and RhoA/ROCK work in a complementary manner to support urokinase-type plasminogen activator-stimulated cell migration [51]. Thus, our results identified an additional pathway activated by DDR1, which is of critical importance for effector T cell migration in 3D collagen.

Our findings also support a role for DDR1 in Th17 chemotaxis in 3D collagen. Recently, it has been reported that IL-8- and LTB-4-induced chemotaxis of neutrophils in 3D collagen involves DDR2-mediated proteolysis *via* the secretion of MMP-8 [30]. However, DDR1-mediated Th17 chemotaxis in 3D collagen occurred independently from MMP activity. Therefore, the implication of MMPs in chemotaxis could be leukocyte type-dependent.

Using the mouse dorsal air pouch model of leukocyte migration, we provided, to our knowledge, the first evidence that DDR1 is implicated in T cell migration *in vivo*. Collagen is abundant in the skin, and to reach the air pouch, T cells must migrate through the endothelial cell layer and overcome the collagen barrier of the extravascular tissue. Thus, the DDR1:Fc blocking receptor reduced the number of Th17 cells in the air pouches likely by preventing them from migrating across collagen. These results support the findings with human polarized Th17 cells suggesting that the function of DDR1 in these cells is not simply due to the *in vitro* differentiation and activation. Accordingly, DDR1 may play an important biological role by facilitating the migration and the positioning of effector T cells in collagen-rich tissues during adaptive immune response and autoimmune diseases. This could be the case during chronic arthritis or psoriasis, which are associated with the recruitment of Th17 cells into the joints and skin and where collagen is a predominant matrix protein. Therefore, blocking DDR1 could represent a novel therapeutic strategy in these diseases and other connective tissue diseases driven by Th17 cells. However, additional work is required to investigate the importance of DDR1 in different biological systems.

DDR1 inhibition decreased the recruitment into the air pouch of not only Th17 cells but also of non-Th17/CD4^+^ T cells. The human polarized Th17 cells used in this study contain Th1 cells [52] and our results suggest that these cells also depend on DDR1. These data indicate that DDR1 could also be important for the migration of other T helper subsets.

In summary, we have shown that DDR1 is a key pathway in the migratory function of Th17 cells. The calreticulin-thrombospondin-1 interaction has been described as an autocrine pathway regulating T cell migration in 3D collagen [53]. Whether there is a crosstalk between these two pathways is unclear. Further understanding of how DDR1 stimulates T cell migration in collagen and in extravascular tissues will likely lead to novel insights into the development of the adaptive immune response, inflammatory diseases and metastasis.

## MATERIALS AND METHODS

### Reagents and antibodies

X-vivo 15 medium and the human T cell nucleofector kit were from Lonza technologies (Basel, PMA, ionomycin, collagenase IV, ROCK inhibitor (Y27632) and the general MMP inhibitor (GM6001) were from Sigma-Aldrich (St. Louis, MO). Human cytokines (TGF-β, IL-1β, IL-6, IL-23), the chemokine CCL20, the recombinant human and mouse blocking receptors DDR1:Fc and their respective isotypic controls, the recombinant human and mouse IgG-Fc fragments (control IgGs) were from R&D Systems (Minneapolis, MN). The human naïve CD4^+^ T cell isolation kit was from STEMCELL Technologies (Vancouver, BC). Rat-tail type I collagen was from Corning (Bedford, MA). Anti-mouse CD4-FITC (clone RM4-5), anti-human CCR6-Alexa 647 (clone 11A9), anti-mouse IL-17-Alexa 647 (clone TC11-18H10), anti-human IL-17-Alexa 647 (clone N49-653), and control antibodies were from BD Biosciences (San Diego, CA). Human anti-DDR1-PE (clone 51D6) was from Biolegend (San Diego, CA). Non-conjugated rabbit anti-mouse and human DDR1 (clone C-20), non-conjugated rabbit anti-human DDR2 (clone H108) antibodies and the anti-phospho-ERK1/2 (clone E-4), anti-ERK2 (clone C-14), and anti-β-actin (clone C-2) Abs were from Santa Cruz Biotechnology (Santa Cruz, CA). CD3/CD28 Dynabeads were from Invitrogen Dynal AS (Oslo, Norway). C3 toxin (RhoA inhibitor) and G-LISA kits for RhoA and Rac1 activities were from Cytoskeleton Inc (Denver, CO). ROCK Activity Assay was from Cell Biolabs (San Diego, CA). The Rac1 inhibitor NSC 23766 was from Tocris Bioscience (Bristol, UK). Calcein-AM and the MAPK/ERK inhibitor PD98059 were from Calbiochem (San Diego, CA).

### Human Th17 cells

Human naïve CD4^+^ T cells were isolated from peripheral blood of healthy adult volunteers according to the requirements of and with the approval of the Laval University ethical committee. The cells were then polarized towards Th17 cells as we previously described [54].

### DDRs expression, IL-17 intracellular staining and flow cytometry

To identify IL-17 producing cells, human polarized Th17 cells were activated or not with PMA (50 ng/ml) and ionomycin (1 μM) (6 h at 37°C) in the presence of 5 μg/ml GolgiPlug (BD Biosciences) containing brefeldin A to interfere with cytokine secretion. The cells were first stained with PE-conjugated extracellular anti-DDR1 antibody, washed, fixed and permeabilized with a CytoFix/CytoPerm kit and stained with intracellular Alexa-647-conjugated anti-human IL-17 antibody. To detect DDR2 expression, permeabilized cells were stained with rabbit intracellular anti-human DDR2 antibody followed with PE-conjugated anti-rabbit IgG secondary antibody. The cells were washed and stained with intracellular anti-IL-17-Alexa-647 antibody. After staining, the cells were washed and analyzed by flow cytometry (BD FACSCalibur II). Cells stained with isotypic antibodies were used as controls.

### Collagen gel and coating

Collagen gels were prepared by diluting rat-tail type I collagen to a final concentration of 1.65 mg/ml in X-vivo medium and adjusted to pH7.4 with NaOH. The cells were mixed with this solution and the suspension was allowed to polymerize (1 h at 37°C) in 8 well-Lab-Tek plates or in 24 well-plates, respectively for cell migration and signaling experiments.

For collagen coating (2D), rat-tail type I collagen was diluted in PBS to 100 μg/ml and 300 μl of the solution was added to 24 well plates. The plates were incubated for 2 h at 37°C, after which they were washed three times with PBS before seeding the cells.

### Cell migration in 3D collagen

Th17 cells were embedded in collagen gels and their motility was evaluated by live cell confocal microscopy. As indicated and before mixing the cells with collagen, 20 μg/ml of recombinant human DDR1:Fc or control human IgG (recombinant Fc fragment) were added to the collagen solution, which was then incubated on a rotary shaker for 2 h at 4°C. Th17 cells in medium were labelled with calcein-AM (5 nM) for 30 min in the dark at 37°C. After washing with PBS, cell pellets were resuspended in the collagen solution (2 × 10^6^ cells in 300 μl), distributed in 8 well-Lab-Tek plates and incubated at 37°C for 1 h to allow collagen polymerization. The wells were then placed at 37°C in a pre-warmed environmental chamber (LiveCell3, Pathology Devices) and the cells were observed by digital time-lapse using a spinning disk confocal microscope (Wave FX-Borealis-Leica DMI 6000B, Quorum Technologies) and a 10X objective (HC PL Apo NA 0.4). Images were recorded using an Image EM-camera (Hamamatsu photonics) for 30 min with 30 seconds frame intervals. The migratory distance of 100 cells for each sample was quantified by computer-assisted cell tracking (Volocity software, PerkinElmer) and the average speed (velocity) per cell was calculated and expressed as μm/min.

Th17 cell migration in 3D collagen was also performed using transwell inserts of polycarbonate membrane (3 μm, BD Biosciences) coated with collagen gels and mounted in 24-well plates. 30 μl of the collagen solution was overlaid on the inserts, which were then incubated for 1 h at 37°C to allow collagen polymerization. Cell suspensions (5 × 10^5^ cells in 100 μl of X-vivo medium) were then added on top of the collagen gels. After 24 h, cells that had passed through the transwells to the other side of the filters and in the outer wells, containing or not 1 μg/ml of the chemoattractant CCL20, were recovered and counted microscopically by a blinded observer.

### RNA interference

Human Th17 cells were transfected using the Nucleofector^TM^ 2b device (program V-024) and the human T cell Nucleofector kit reagents as recommended by the manufacturer (Lonza technologies, Basel, Cells (5 × 10^6^) cultured for four days in Th17 polarizing conditions were transfected with 200 nM of DDR1 specific siRNAs (HSS1878780) and (HSS187879) or of control non-silencing siRNA (Invitrogen). After nucleofection, the cells were immediately transferred to pre-warmed X-vivo medium and incubated for 6 hours. Live cells were then recovered by Ficoll separation and cultured for an additional 42 hours before being used in subsequent experiments. The efficiency of DDR1 silencing was assessed by flow cytometry and western blot.

### Plasmids and cell transfection

The plasmid encoding the dominant-negative form of MEK-1 (DN-MEK-1) was described in our previous studies [55, 56]. Human Th17 cells were transfected with 5 μg of flag-DN-MEK-1 and control pcDNA plasmids using the nucleofector method as described for siRNA.

### ELISA assays for RhoA/ROCK, Rac1 and DDR1 tyrosine phosphorylation

GTP-bound RhoA and Rac1, and ROCK activity were determined by previously used commercial ELISA kits [57-59]. Th17 cells were activated with immobilized collagen (100 μg/ml) or embedded in collagen gels (1.65 mg/ml) for 1 h at 37°C. The cells were released from collagen gels by a treatment with collagenase IV (1 mg/ml for 30 min at 37°C). Cells in contact with immobilized collagen (2D) were also treated with collagenase. Equal cell numbers were lysed using RIPA buffer, and equal amounts of proteins for each sample were then assayed for RhoA, Rac1 and ROCK activation as recommended by the manufacturers.

The DDR1 tyrosine kinase activity was measured with the previously used PathScan Phospho-DDR1 (pan-Tyr) Sandwich ELISA kit [60] (Cell Signaling Technology, Beverly, MA) according to the manufacturer's instructions.

### Western blot analysis

DDR1 protein and ERK phosphorylation levels were determined by western blot analysis using anti-DDR1 (C20) and anti-phosphorylated ERK1/2 (clone E-4) antibodies as we previously described [54]. The blots were stripped and reprobed with control antibodies.

### Air pouch model and Th17 migration *in vivo*

All procedures involving animals were conducted according to the requirements of and with the approval of the Laval University animal protection committee. Th17 recruitment *in vivo* was carried out as previously described [36]. Briefly, air pouches were raised on the dorsum of 6-8 weeks old female C57BL/6 mice (Jackson Laboratories) by injection of 3 ml sterile filtered air on days 0 and 3. On day 6, 1 ml of PBS containing CCL20 (400 ng) was injected into the pouches to induce Th17 recruitment. Where indicated, 50 μg of blocking recombinant mouse receptor DDR1:Fc or control mouse recombinant IgG-Fc (Fc fragment) were injected into the air pouches at the same time as CCL20. 24 h later, mice were sacrificed and air pouches washed twice with cold PBS. The resulting cell suspensions were activated with PMA+ionomycin in the presence of brefeldin A, stained with anti-CD4-FITC mAb, fixed/permeabilized and incubated with rabbit anti-mouse DDR1 (C20) followed with PE-anti-rabbit IgG secondary antibody. The cells were washed, stained with intracellular anti-mouse IL-17-Alexa-647 antibody, washed and analyzed by flow cytometry.

### Statistical analysis

Statistical analysis were performed by the Student's t-test. Results with p < 0.05 were considered significant.

## Supplementary Material and Figures




