# Supplementary material for: Discoidin domain receptor 1 promotes Th17 cell migration by activating the RhoA/ROCK/MAPK/ERK signaling pathway
Source: Oncotarget. 2016 Jul 6;7(29):44975–90. doi: 10.18632/oncotarget.10455 (PMC5216699; doi:10.18632/oncotarget.10455)
Supplement: Supplementary file 1 [file oncotarget-07-44975-s001.pdf]

Discoidin domain receptor 1 promotes Th17 cell migration by activating the RhoA/ROCK/MAPK/ERK signaling pathway

Supplementary Material

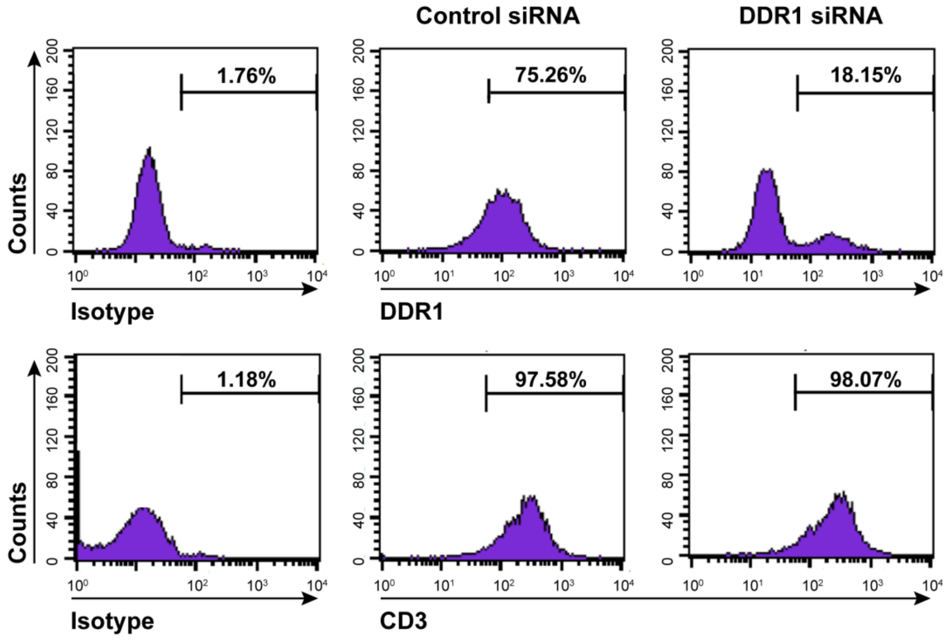

Supplementary Figure S1: DDR1 siRNA (HSS1878780) does not affect the expression levels of the CD3 receptor. Human polarized Th17 cells were transfected with control or DDR1 siRNAs by the nucleofector method as indicated in the "Materials and Methods" section. After 48 h of transfection, the cells were stained with isotypic, anti-DDR1 and anti-CD3 antibodies and analyzed by flow cytometry. Results are representative of five independent experiments performed with Th17 cells derived from five different blood donors.

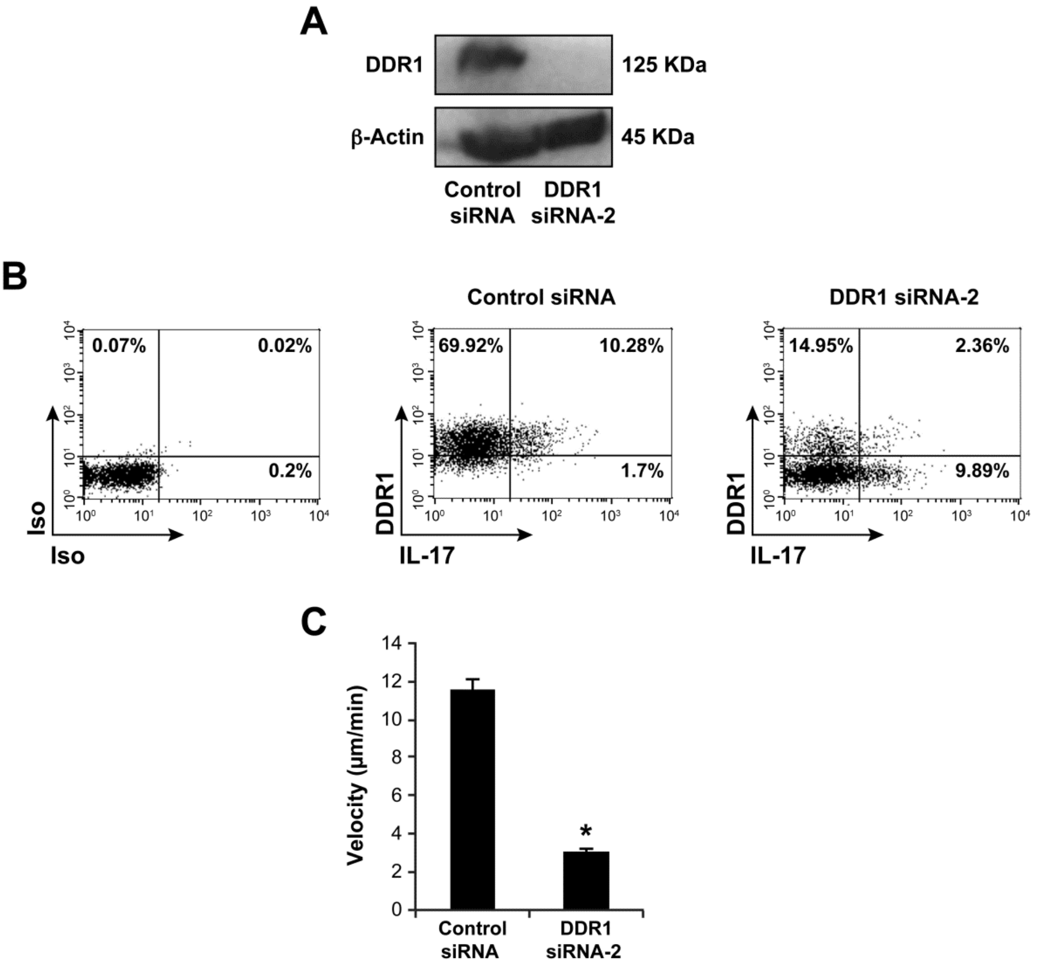

Supplementary Figure S2: An additional DDR1 siRNA sequence (HSS187879) (DDR1 siRNA-2) inhibits Th17 migration in 3D collagen. CD4$^+$ T cells cultured for four days in Th17 polarizing conditions were transfected with 200 nM of DDR1 siRNA-2 or of control non-silencing siRNA (Invitrogen). The DDR1 levels were assessed by western blot (**A**) and by flow cytometry after reactivating the cells with PMA+ionomycin to identify IL-17-producing cells (**B**). (**C**) Th17 transfected with DDR1 siRNA-2 or control siRNA were embedded in collagen gels and cell motility was quantified by live cell confocal microscopy. The results are mean values ± SD of three independent experiments performed with Th17 cells derived from three different blood donors. *p <0.05.

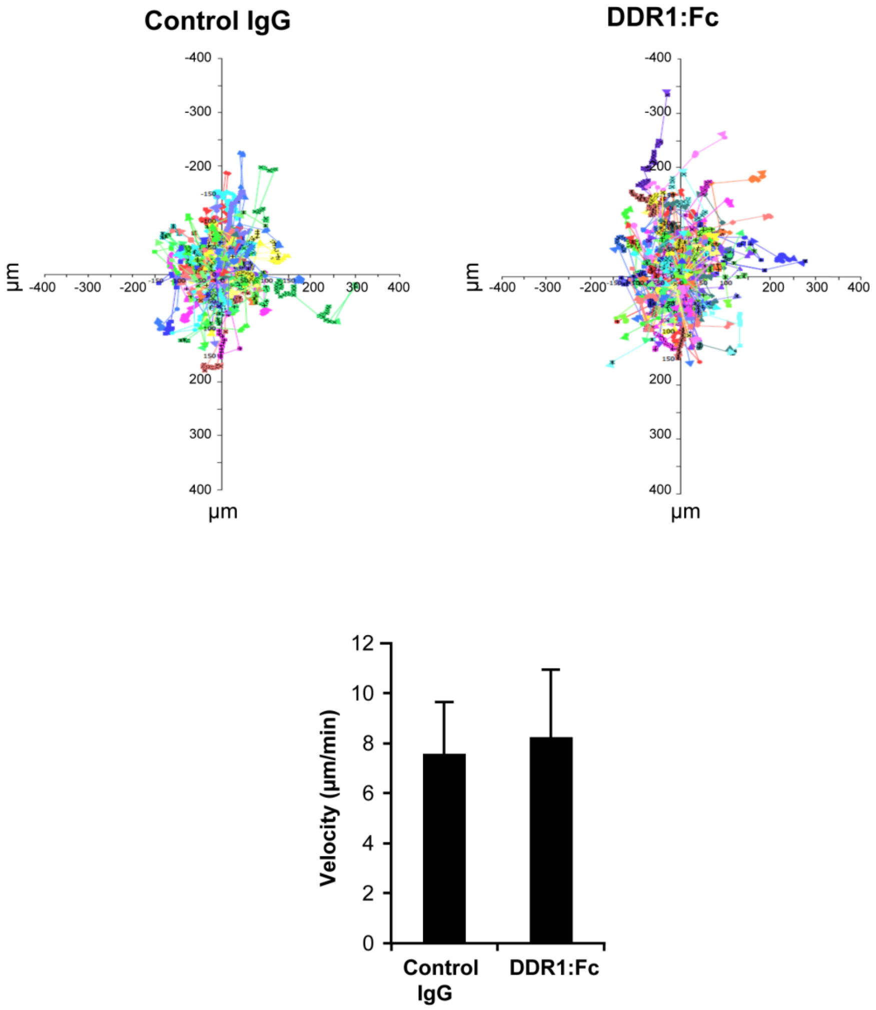

Supplementary Figure S3: DDR1:Fc has no effect on neutrophil migration in 3D collagen. Neutrophils were isolated from human peripheral blood and embedded in collagen gels containing either control IgG or DDR1:Fc blocking receptor. Neutrophil migration was then monitored by live cell confocal microscopy and the velocity of movement was determined as described in the "Materials and Methods" section. Results are mean values ± SD of three independent experiments performed with neutrophils isolated from three different blood donors.

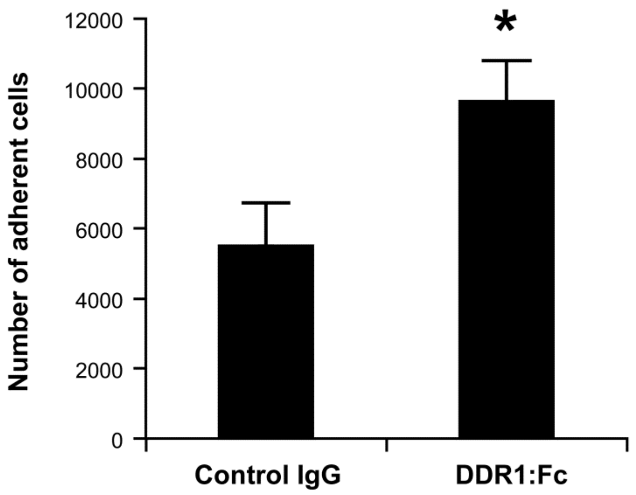

Supplementary Figure S4: Polarized Th17 cells cultured in collagen gel containing DDR1:Fc display enhanced adhesion to collagen. The cells were cultured for 1 h in collagen gels containing either control IgG or DDR1:Fc. They were then released from collagen gels and seeded in collagen-coated wells. After 1 h, the cells in suspension were washed and adherent cells were detached through three different washes and counted microscopically. Results are mean values ± SD of three independent experiments performed with Th17 cells derived from three different blood donors. **p* <0.05.

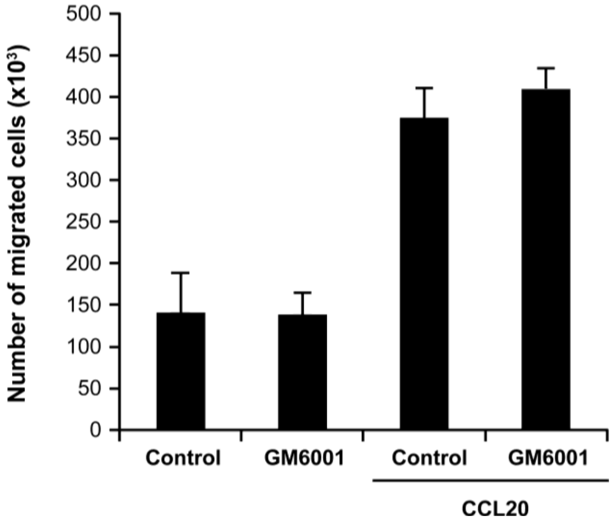

Supplementary Figure S5: Polarized Th17 cells migrate in 3D collagen independently from matrix metalloproteinases (MMP). Polarized Th17 cells were incubated for 1 h with diluent (control) or 50 μM of GM6001 (MMP inhibitor) and then placed on top of transwells coated with collagen gels containing diluent (control) or 50 μM of GM6001. The cells that had passed to the outer wells containing medium alone or with CCL20 were counted microscopically. Results are mean values ± SD of three independent experiments performed with Th17 cells derived from three different blood donors. Similar results were obtained when cell migration was assessed by confocal microscopy (data not shown).

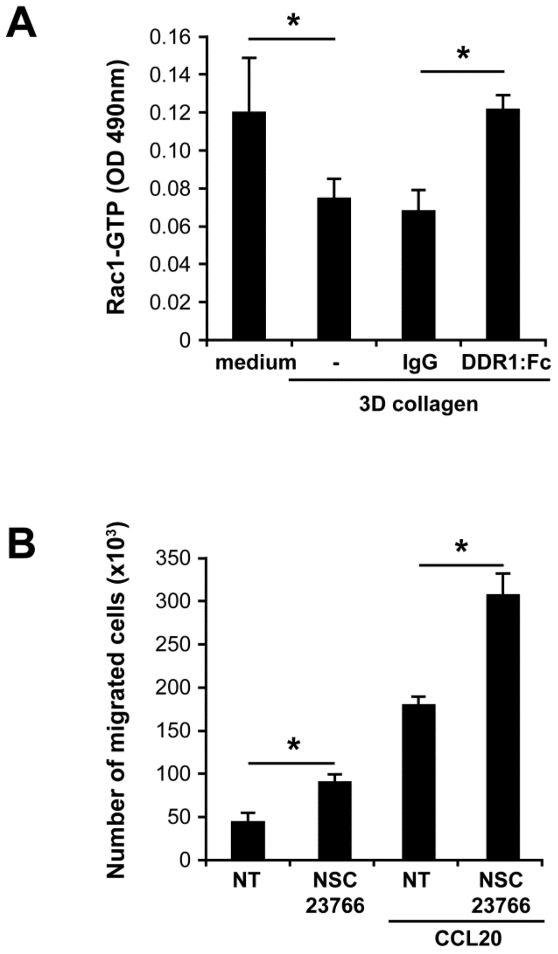

Supplementary Figure S6: DDR1 promotes Th17 migration in 3D collagen by reducing Rac1 activity. **(A)** DDR1 blockade enhances Rac1 activation by collagen gel. Human polarized Th17 cells were cultured in medium or embedded in collagen gels containing DDR1:Fc or control IgG. The cells were released from collagen, lysed and Rac1 activity was determined using the Rac1 specific ELISA. **(B)** The Rac1 inhibitor enhances Th17 migration in 3D collagen. The cells were treated or not (NT) with 20 μM of NSC 23766 (Rac1 inhibitor) for 1 h and tested for their invasion capacity of collagen gel-coated transwells. After 24 h, the cells that had passed to the outer wells containing medium alone or with CCL20 were counted microscopically. Results (panels A and B) are mean values ± SD of three independent experiments performed with Th17 cells derived from three different blood donors. *p <0.05.

Video 1: DDR1 siRNA inhibits Th17 motility in 3D collagen. Polarized human Th17 cells were transfected with control or with DDR1 siRNAs. After transfection, the cells were labelled with calcein AM (5 nM) and embedded in collagen gels. Cell migration was evaluated by live cell confocal microscopy as described in the “Materials and Methods” section. The recordings showing the migration of control siRNA- (left) or DDR1 siRNA-transfected cells (right) are representative of three independent experiments performed with Th17 cells derived from three different blood donors.
